# Investigating factors that influence residency program selection among medical students

**DOI:** 10.1186/s12909-023-04602-9

**Published:** 2023-08-29

**Authors:** Saud Abdulaziz Alaqeel, Bader Khalid Alhammad, Suhail Mohsen Basuhail, Khayyal Mohammed Alderaan, Abdulla Taher Alhawamdeh, Mohammed Fahad Alquhayz, Alwaleed Mansour Alzunaidi, Fahad Abdulaziz Alrashed

**Affiliations:** 1 Collage of Medicine, King Saud University, Riyadh, Saudi Arabia; 2 Department of Cardiac Sciences, College of Medicine, King Saud University (KSU), P.O. Box 7805, Riyadh, 11472 Saudi Arabia

**Keywords:** Medical education, Students Assessment, Residency Program, Health care, Medical students

## Abstract

**Backgrounds:**

Medical students and interns face several decisions during their career development. One of the most critical decisions they have to make is choosing a residency program that suits their career goals and aspirations. The selection process can be challenging, as several factors can influence the decision-making process. There was limited research on the role of GPA and opportunities in residency program selection. Therefore, this study examines the factors that influence interns’ and 5th-year medical students’ choices of residency programs.

**Methods:**

Observational and analytical cross-sectional studies were conducted at King Saud University (KSU) in Riyadh, Saudi Arabia. A sample of 5th-year medical students as well as medical interns was included in the study. This study only includes students who were interested in choosing a residency program in one of these eight domains: Medicine, Family Medicine, Orthopedics, Pediatrics, Radiology, Surgery, Obstetrics/Gynecology, and Ophthalmology.

**Results:**

The total number of students with 5th-year medical students was 205 (60.1%) and the number of students with medical interns was 135 (39.5%). The majority of students (51.0%) had a GPA above 4.5 out of 5, followed by 3.5 to 4.5 among 146 (42.8) students, and only 21 (6.2) had a GPA below 3.5. Those students with less than a 3.5 GPA out of 5 were found not to have any favorite residency program specialties when it came to academic achievement. Furthermore, in the case of those students whose GPA was 3.5 to 4.5, they had a certain mindset when it came to choosing a residency program. A 1.9 times greater likelihood of selecting obstetrics and gynecology (OR = 1.9, p = 0.19) was achieved by students with GPAs between 3.5 and 4.5, followed by a 1.5 times greater probability of selecting radiology (OR = 1.5; p = 0.55). High achievers, ophthalmology (OR = 1.7; p = 0.32) and surgery (OR = 1.4; p = 0.31) were the most popular residency programs. A student with a high GPA (mean score = 7.3) also chooses his or her career based on better opportunities than a student with a low GPA (mean score = 6.8).

**Conclusions:**

The study highlights the correlation between academic achievement and preferred specialties for future careers among medical students. While the study’s findings should be considered in the context of various other factors such as lifestyle, social life, and income can influence students’ preferred specialties for residency programs.

## Introduction

Medical students and interns face several decisions during their career development. Selecting a residency program that fits their career goals and aspirations is one of the most critical decisions they have to make. The process of choosing a residency program specialty that will guide careers and impact personal lives was complicated and multifaceted. Multiple factors can influence the decision-making process during the selection process. The factors that influence the selection of a residency program were critical to improving the match between residents and their programs, reducing the attrition rate, and improving the quality of care. Research has been conducted throughout the world, including in the USA [1], Brazil [2], Canada [3], and Japan [4], to analyze the factors affecting medical students’ choice of specialty worldwide. For instance, in a study, the authors found that program location and the availability of subspecialty training were significant factors in the selection of a residency program [5]. Similarly, studies found that the availability of research opportunities, program reputation, and work-life balance were critical in influencing the selection process [6, 7]. In a study, the authors found that the quality of life, the cost of living, and the availability of recreational activities in the location of the residency program were significant factors in the selection process [8]. Lifestyle issues have become an increasingly important factor in guiding potential residents in choosing a career path as they make a career choice [9, 10]. The authors also found that the availability of mentors, social support, and the opportunity to work in a rural or underserved area were critical in influencing the selection process. It has been observed that demographics, interest, lifestyle, finances, and prestige can influence residency choice, but because these studies were conducted at a single time point, we cannot determine how these factors may change over time [9, 11]. Furthermore, a study found that the availability of exposure to various medical specialties, a supportive work environment, and the potential for financial stability were critical in the selection process [11]. In contrast, a study found that program reputation, work-life balance, and the availability of subspecialty training were significant factors in the selection process [12]. Medical students and interns have studied factors that influence the choice of a residency program. Studies have identified factors such as the location of the program, its prestige, and its reputation as important considerations in selecting residency programs [13, 14]. Similarly, program attributes, including educational curriculum, research opportunities, and work-life balance, were critical in influencing the selection of a residency program [11]. According to the findings of a study carried out at Kuwait University, the most influential factor in choosing a specialty is to observe good treatment outcomes followed by working in a specialty that requires a high level of skill, according to Kuwaiti students who took part in the study [15]. To deal with the decreasing number of students interested in primary care (PC) as a career path, a number of factors associated with choosing PC have been extensively studied [11, 16]. It has been reported in some studies that students with high GPAs were more likely to select a residency program that provides them with the best career prospects [4, 17]. Evidence suggests that a student’s GPA was strongly correlated with positive performance when selecting a residency program or performing during residency [18, 19]. Although extensive research has been conducted on factors that influence residency program selection. There was limited research on the role of GPA and opportunities in residency program selection among Saudi medical students and interns. Therefore, this study examines the factors that influence residency program choice by both interns and fifth-year medical students.

## Methods

### Data collection

Participants will be asked to complete an online Google form survey questionnaire that will be sent to them via e-mail as well as hard copy. Observational and analytical cross-sectional studies were conducted at King Saud University (KSU) in Riyadh. It was the first and largest medical college in Riyadh, Saudi Arabia. Over five months were spent conducting the study, starting in November 2022 and ending in March 2023. A sample of 5th-year medical students as well as medical interns was included in the study. This study only includes students who were interested in choosing a residency program in one of these eight domains: Medicine, Family Medicine, Orthopedics, Pediatrics, Radiology, Surgery, Obstetrics/Gynecology, and Ophthalmology. Except for these eight, all other medical residency programs were excluded. Students in other years of medical school and those in residency programs were excluded. The sample size was calculated using the formula n = Z _1-a/2_^2^ (SD) ^2^ / d^2^, where Z _1-a/2_^2^ = standard normal variate (at 5% type 1 error (P < 0.05) it is 1.96 and 1% type 1 error (P > 0.01) it was 2.58). In our case, P values were considered significant below 0.05; hence 1.96 was used in the formula. d = Absolute error 5%. SD = Expected residents and programs selection by students based on previous, current and pilot studies (49%, 33%, 32% and 29%) [n = 1.96^2^ * (49)^2^ / 5^2^ = 368.7]. The calculated sample size was 368, however sample size of our study was much closer to what we expected (n = 341). The study was approved by the Institutional Review Board at the College of Medicine, King Saud University.

### Questionnaire setting

A self-administered questionnaire was developed by members of the research team following a comprehensive literature review. Following the development of the questionnaire, we send these questionnaires to the questionnaire community under the Ethical board section of the questionnaire committee. It was worth noting that the members of the questionnaire community have expert knowledge of the different medical and educational fields. There were 21 items in the prepared version, all of which had been subjected to detailed discussion among members of a questionnaire committee as a whole. There were only eight items (with sub-domain) agreed upon after a revision of the questionnaire was made in two meetings. We carefully revised the questionnaire to ensure it met the study’s objectives. The questionnaire has three parts. The first part of the form was demographic information, which includes age, gender, marital status, year of study, and GPA. As part of the second part of the exam, we target only those students who select the following eight major career paths: Medicine, Orthopedics, Pediatrics, Radiology, Surgery, Obstetrics/Gynecology, Family medicine, and Ophthalmology. Moreover, they were asked to rank their top career choices and indicate with a “yes” or “no” which career they believed was possible. Students were asked to rate 19 variables based on the degree to which they influenced their decision to choose a residency program, using a Likert scale of 1 to 5 (no impact to major impact). A pilot study for validation of the questionnaire was recommended by the consulted team, as well as the ethical team. We conducted a pilot study with 17 participants about influenced factor about selection of residency program questionnaire. A Cronbach’s alpha reliability of 0.836 was found for all eight questions and subdomains in the instrument.

### Data analysis

The quantitative and categorical variables were described using descriptive statistics (frequency distributions and percentages) based on the frequencies. For statistical testing using the chi-square, odd ratio, and principal components factor analysis based on the GPA, the analysis was conducted by using the chi-square and principal components factor analysis. A p-value of < 0.05 was calculated to report the statistical significance of the results. Data were analyzed using SPSS program 24.

## Results

We approached 383 5th-year and internship students for the current study. However, 341 (89.03%) students agreed to participate in this study. There were 231 participants in this study (67.7%) who were male and 110 participants (32.3%) who were female. The average age of participants was 24.3 + 1.4, and the majority (331, 97.1%) were Saudi nationals. The total number of students with 5th-year medical students was 205 (60.1%) and the number of students with medical interns was 135 (39.5%). The majority of students (51.0%) had a GPA above 4.5 out of 5, followed by 3.5 to 4.5 among 146 (42.8) students, and only 21 (6.2) had a GPA below 3.5. Most of the students had some favorite specialties in the health field (Table 1). In the tables (Table 2) showed that students favorites specialties to select future career. Most of the students want medicine (26.4%) as a health specialty as a favorite, as followed surgery (19.9%), family medicine (11.7%), then orthopedics (9.7%), pediatrics (8.5%) as so on. However, 13.8% students not reported any specialties (residency program). Medical students and interns in Saudi Arabia were mostly interested in pursuing medicine specialties.

**Table 1:** Demographic information of participants

Items	Categories	Not tested or reported	n(%)
Gender	Male		231(67.7)
	Female		110(32.3)
Age	20–23	0	109(32.0)
	24–26	0	224(65.7)
	27- or more	0	8(2.3)
Nationality	Non- Saudi		10(2.9)
	Saudi		331(97.1)
Year of study	5th year	1	205(60.1)
	Medical intern		135(39.6)
GPA	less than 3.5		21(6.2)
	3.5–4.5		146(42.8)
	More than 4.5		174(51.0)
Are you some favorite specialties	Yes		296(86.8)
	No		45(13.2)

**Table 2:** Discipline wise residential program selection by the 5 year and intern students

Variables	n(%)
Surgery	68(19.9)
Medicine	90(26.4)
Orthopedics	33(9.7)
Pediatrics	29(8.5)
Obstetrics & Gynecology	17(5.0)
Family Medicine	40(11.7)
Radiology	7(2.1)
Ophthalmology	10(2.9)
Not reported or missing	47(13.8)

Students’ favorite specialties for future careers and their academic achievement during full medical education were correlated in Table 3. Those students with less than a 3.5 GPA out of 5 were found not to have any favorite residency program specialties when it came to academic achievement. One possible explanation could be that students with lower GPAs may not have had the same level of exposure or opportunities to explore different residency specialties. Students with lower GPAs may have dedicated more time and effort to improving their grades, leaving less time for exploring different specialties. Furthermore, in the case of those students whose GPA was 3.5 to 4.5, they had a certain mindset when it came to choosing a residency program. A 1.9 times greater likelihood of selecting obstetrics and gynecology (OR = 1.9, p = 0.19) was achieved by students with GPAs between 3.5 and 4.5, followed by a 1.5 times greater probability of selecting radiology (OR = 1.5; p = 0.55). Those with a GPA between 3.5 and 4.5 were also most likely to choose medicine (OR = 1.2; p = 0.65) and pediatrics (OR = 1.4; p = 0.47) as their residency program in the current study. Moreover, Students with GPAs over 4.5 were considered high achievers. Among high achievers, ophthalmology (OR = 1.7; p = 0.32) and surgery (OR = 1.4; p = 0.31) were the most popular residency programs, followed by orthopedics (OR = 1.3; p = 0.5) and medicine (OR = 1.0; p = 0.89). Students with GPAs greater than 4.5 were considered high achievers and were well-suited to careers in ophthalmology and surgery. Their exceptional academic performance, coupled with their analytical skills, attention to detail, and ability to handle pressure, makes them ideal candidates for these demanding specialties. Furthermore, their dedication to continuous learning and passion for helping others.

**Table 3:** Students’ favorite specialties for future careers associated with their academic achievement during full medical education

Variables		GPA less than 3.5			GPA 3.5–4.5				GPA more than 4.5	
	n(%)	n(%)	OR (95% CI)	*p*	n(%)	OR (95% CI)	*p*	n(%)	OR (95% CI)	*p*
Surgery	68(19.9)	2(9.5)	0.09(0.02–0.45)	0.003	22(15.1)	0.84(0.40–1.7)	0.64	44(25.3)	1.4(0.73–2.6)	0.31
Medicine	90(26.4)	6(28.6)	0.22(0.08–0.62)	0.004	40(27.4)	1.2(0.60–2.2)	0.65	44(25.3)	1.0(0.56–1.9)	0.89
Orthopedics	33(9.7)	2(9.5)	0.20(0.04–0.95)	0.04	11(7.5)	0.87(0.36- 2.0)	0.75	20(11.5)	1.3(0.61–2.7)	0.5
Pediatrics	29(8.5)	1(4.8)	0.11(0.01–0.92)	0.04	15(10.3)	1.4(0.59- 3.0)	0.47	13(7.5)	0.95(0.41–2.1)	0.91
Obstetrics & Gynecology	17(5.0)	1(4.8)	0.19(0.02–1.6)	0.13	12(8.2)	1.9(0.73–4.6)	0.19	4(2.3)	0.50(0.15–1.6)	0.26
Family Medicine	40(11.7)	2(9.5)	0.16(0.03–0.78)	0.02	22(15.1)	1.4(0.67- 3.0)	0.34	16(9.2)	0.85(0.39–1.84)	0.68
Radiology	7(2.1)	0(0)	NA		4(2.7)	1.5(0.38–5.7)	0.55	3(1.7)	0.91(0.21–3.8)	0.9
Ophthalmology	10(2.9)	0(0)	NA		2(1.4)	0.52(0.10–2.6)	0.42	8(4.6)	1.7(0.57–4.9)	0.32
Not reported or missing	47(13.8)	14(66.7)	Ref-1		18(12.3)	Ref-1		22(12.6)	Ref-1	

A table describing the factors affecting career choice can be found in Table 4. Students with high achievement chose residency programs because of the challenging specialty, which showed significant differences between the two groups (p = 0.003) compared with less than 4.5 GPA achievers. Students with high achievement were drawn to residency programs in challenging specialties for several reasons. Their motivation to continuously excel, passion for their chosen specialty, and the opportunity to receive comprehensive training all contribute to their preference for these programs. The significant differences observed between students with high GPAs and those with lower GPAs underscore the distinct career paths chosen by these two groups. A student with a high GPA (mean score = 7.3) also chooses his or her career based on better opportunities than a student with a low GPA (mean score = 6.8). A student’s GPA plays a crucial role in shaping their career opportunities. A high GPA reflects dedication, intellectual capability, and problem-solving skills, making students more attractive to employers and prestigious educational institutions. Conversely, a low GPA may limit career options and hinder access to financial aid and educational opportunities. As compared to higher GPA students, low GPA students scored higher for these factors for career choices: good social life (6.9), working hours after residency (6.8), high income (6.2), lifestyle during residency (6.9), and research opportunities (6.4). It was found that the mean score of high GPA students was significantly higher than that of low GPA students in terms of influencing factors concerning residency programs, good treatment outcomes (p = 0.0001), learning and academic opportunities (p = 0.03), good reputation and prestige (p = 0.0001), doctor-patient relationships (0.0001), and emergency treatment (p = 0.0001).

**Table 4:** Describing the factors affecting career choice

Influencing factors	More than 4.5 GPA (Mean Score)	Less than 4.5 GPA * (Mean Score)	p-values
Challenging specialty	6.9	5.3	0.003
Better Opportunities	7.3	6.8	0.06
Like every one known me best doctor	6.4	6.3	0.18
Experience on Core Rotation	6.5	5.9	0.009
Good social life	6.7	6.9	0.61
Work Hours After Residency	6.3	6.8	0.21
Good treatment outcomes on my patients with Patient Relationships	7.2	6.3	0.0001
Learning and academic Opportunities	6.4	5.9	0.03
I want a high income After Training	5.8	6.2	0.008
Future Patient Demographics	5.3	5.5	0.09
Good reputation and prestige	5.1	4.2	0.0001
Ability to Obtain Residency Position	6.8	5.7	0.0001
Lifestyle During Residency	6.4	6.9	0.01
Length of Training Required	5.4	5.4	0.4
Work Hours During Residency	4.8	5.6	0.003
Acceptable on-call duty and call Schedule	4.3	4.7	0.002
Treat emergency cases	6.4	4.9	0.0001
More research opportunities	5.8	6.4	0.005
Enjoyed the specialty during my study.	5.9	5.7	0.2

* We add (less than 3.5) + (3.5 to 4.5) as a less than 4.5 GPA

Table 5 showed the demographic information with student’s selection of their residence program specialties. Significant differences found in the gender regarding the selection of residence program, among these male students were more likely to select surgery (77.9%), medicine (67.8%), orthopedics (93.9%) and ophthalmology (100%) as compared to the female students. Similarly, majority of 5th-year medical students wanted to choose surgery (60.3%) over medical interns (39.7%), and most also wanted to study family medicine (72.5%). Furthermore, higher GPA students chose surgery (64.7), orthopedics (60.6%), medicine (48.9%), and ophthalmology (80.0%) over other ranks of GPA. The current results highlight the career preferences and specialty choices of 5th-year medical students. The majority of students indicated a preference for surgery over medical internships, while also displaying a significant interest in family medicine. Moreover, the findings suggest a correlation between higher academic performance and preferences for surgery, orthopedics, medicine, and ophthalmology.

**Table 5:** The demographic information with student’s selection of their residence program specialties

Variables	categories	n(%)	Surgery	Medicine	Orthopedics	Pediatrics	Obstetrics & Gynecology	Family Medicine	Radiology	Ophthalmology	Chi-square (*p*-value)
Age	20–23	109(32.0)	21(30.9)	32(35.6)	8(24.2)	9(31.0)	3(17.6)	15(37.5)	3(42.9)	3(30.0)	13.38(0.49)
	24–26	224(65.7)	45(66.2)	55(61.1)	25(75.8)	20(69.0)	12(70.6)	25(62.5)	4(57.1)	7(70.0)	
	27 and more	8(2.3)	2(2.9)	3(3.3)	0(0.0)	0(0.0)	2(11.8)	0(0.0)	0(0.0)	0(0.0)	
Nationality	Saudi	331(97.1)	66(97.1)	87(96.7)	32(97.0)	28(96.6)	17(100)	40(100)	7(100)	10(100)	2.45(0.93)
	Non-Saudi	10(2.9)	2(2.9)	3(3.3)	1(3.0)	1(3.4)	0(0.0)	0(0.0)	0(0.0)	0(0.0)	
Gender	Male	231(67.7)	53(77.9)	61(67.8)	31(93.9)	11(37.9)	8(47.1)	19(47.5)	4(57.1)	10(100)	40.7(0.000)
	Female	110(32.3)	15(22.1)	29(32.2)	2(6.1)	18(62.1)	9(52.9)	21(52.5)	3(42.9)	0(0.0)	
Marital Status	Married	11(3.2)	4(5.9)	3(3.3)	0(0.0)	0(0.0)	0(0.0)	3(7.5)	0(0.0)	0(0.0)	6.70(0.46)
	Not Married	330(96.8)	64(94.1)	87(96.7)	33(100)	29(100)	17(100)	37(92.5)	7(100)	10(100)	
Year	5th year	205(60.1)	41:(60.3)	52(57.8)	17(51.5)	15(51.7)	7(41.2)	29(72.5)	3(42.9)	4(40.0)	8.47(0.29)
	Medical intern	135(39.6)	27(39.7)	38(42.2)	16(48.5)	14(48.3)	10(58.8)	11(27.5)	4(57.1)	6(60.0)	
GPA	less than 3.5	21(6.2)	2(2.9)	6(6.7)	2(6.1)	1(3.4)	1(5.9)	2(5.0)	0(0.0)	0(0.0)	19.53(0.14)
	3.5–4.5	146(42.8)	22(32.4)	40(44.4)	11(33.3)	15(51.7)	12(70.6)	22(55.0)	4(57.1)	2(20.0)	
	More than 4.5	174(51.0)	44(64.7)	44(48.9)	20(60.6)	13(44.8)	4(23.5)	16(40.0)	3(42.9)	8(80.0)	

## Discussion

There have been many changes in medical specializations in Saudi Arabia in recent years. It was widely known that Saudi Vision 2030, an ambitious reform plan for the economy, health, education, infrastructure, recreation, and tourism sectors, was to be implemented. Integrated and continuous healthcare services will be improved through this plan. It was therefore expected that residency positions would increase in every medical discipline in the future [20]. The choice of a medical specialty has a significant impact on the healthcare workforce. The number of physicians available in different specialties was a significant indicator of future demand. DeWitt et al. [21] and Zeldow et al. [22] discovered that 45–70% of medical students continue in their preferred specialty after they graduate from medical school. The current study aimed to investigate the career preferences of 5th-year medical students and medical interns. As previously reported, demographic preferences were taken into account when switching specialty preferences. Many studies have demonstrated that lifestyle and socio-demographic preferences influence students’ choice of specialties; the socio-demographic profile was indeed a significant factor to consider [23, 24]. Study participants averaged a little over 24 years of age. There have been some previous studies about the same domain with similar populations [25, 26]. In current study found that, academic performance, the majority of the participants (51.0%) had a GPA above 4.5 out of 5. That was higher in the previously published study [26].

In the current study most of the students want to pursue medicine (26.4%) as their preferred health specialty, followed by surgery (19.9%), family medicine (11.7%), orthopedics (9.7%), pediatrics (8.5%), and others. However, 13.8% of the students did not report any preferred specialties for their future residency program. The findings of this study shed light on the preferences of students regarding their future health specialties. While medicine emerged as the most sought-after choice, followed by surgery, family medicine, orthopedics, and pediatrics, a significant portion of students remains undecided or open to exploring various options. The results were in accordance with previous studies showing that ~ 30% of medical graduates now pursue residency programs in medicine [27, 28]. It has been observed over the years that more and more medical graduates were choosing surgical specialties, such that today 15% of medical graduates were pursuing surgical training [29, 30], In line with our study’s findings. Family medicine, OB/GYN, radiology, orthopedics, and psychiatry were among the other major specialties pursued by graduates. As previously reported [31–33], Since students choose residencies that differ from what they stated they were interested in when they matriculated, the small increase in entry rates into medicine and surgery was consistent with students’ preferences at the time of matriculation. Additionally, the study suggests that medical students with a lower GPA may be less certain about their preferred specialty for future careers. This may be due to a lack of academic confidence or exposure to different medical specialties. On the other hand, high achieving students may have a better understanding of their strengths and interests, leading them to select more competitive specialties. Students with a GPA above 4.5 were considered high achievers in the study. Among these high achievers, ophthalmology (OR = 1.7; p = 0.32) and surgery (OR = 1.4; p = 0.31) were the most popular residency programs, followed by orthopedics (OR = 1.3; p = 0.5) and medicine (OR = 1.0; p = 0.89). Ophthalmology was a branch of medicine that focuses on the diagnosis and treatment of eye disorders. It requires a deep understanding of the complex structures and functions of the eye. High achievers possess the necessary analytical skills and attention to detail to excel in this field. Their ability to maintain a high GPA indicates their aptitude for grasping intricate concepts and applying them effectively. Surgery, on the other hand, was a specialized branch of medicine that involves performing invasive procedures to treat diseases or injuries. The field of surgery demands a strong foundation in basic sciences, critical thinking skills, and the ability to make quick and accurate decisions. Students with GPAs over 4.5 have shown the discipline, focus, and determination necessary to succeed in this field.

Academic achievement and preferred specialties for future careers were correlated among medical students, according to this study. Furthermore, it emphasizes the importance of academic performance when choosing a residency program. By using the results of this study, medical schools and residency programs can better understand the preferences of medical students and tailor their programs accordingly. A similar finding was reported in a previous study published in Ophthalmology and Surgery: residency programs selected high achievers more often [34–37]. The factors influencing students’ career choices. Students with high academic achievement chose residency programs based on challenging specialties, while those with low academic achievement prioritized factors such as good social life, working hours after residency, high income, lifestyle during residency, and research opportunities. High GPA students also scored significantly higher in terms of influencing factors concerning residency programs, such as good treatment outcomes, learning and academic opportunities, good reputation and prestige, doctor-patient relationships, and emergency treatment. The study also found that educational curriculum, research opportunities, and work-life balance were significant factors in the selection process. These findings suggest that residency programs that offer a well-rounded educational experience, ample opportunities for research, and a supportive work environment may attract more potential residents. These factors may be especially critical for interns who were still in the process of selecting their specialty and may require a more comprehensive educational experience to make an informed decision. Significant differences in the selection of residency programs among genders. Male students were more likely to select surgery, medicine, orthopedics, and ophthalmology as compared to female students. Similarly, to previous publications, our findings provide some important insights into medical students’ choice of specialty [38–40]. There were gender differences in medical students’ preferences and choices in specialties, and previous research in other countries also indicated that pediatrics and obstetrics & gynecology were more popular with females, surgery more popular with males, and internal medicine more popular with both genders [41–44]. Fifth-year medical students preferred surgery over medical interns and most also wanted to study family medicine. Furthermore, higher GPA students were more likely to choose surgery, orthopedics, medicine, and ophthalmology as compared to other GPA categories. Overall, the findings from this study suggest that academic achievement and demographic factors such as gender and level of study can influence students’ preferred specialties for residency programs. It was important to provide medical students and interns with career guidance and counseling services to assist them in making informed decisions about their future career paths. The factors influencing career choices among medical students and interns in other contexts and cultures need to be explored further. Because this study only included Saudi Arabian 5th-year and intern students, the results were limited in generalizability. It was possible that respondents did not understand the specialty-specific questions as intended, since there was no evidence of validity. Following a discussion with a member of our research team, we decided to focus on selective residency programs first since most healthcare industries were experiencing an increase in the number of professionals in these eight key career paths. For this reason, we chose only eight residency programs. However, we will target a wider range of medical disciplines in future research. Furthermore, more research was needed to address this issue given the many factors that contribute to residency selection.

## Conclusion

The study highlights the correlation between academic achievement and preferred specialties for future careers among medical students. While the study’s findings should be considered in the context of various other factors such as lifestyle, social life, and income can influence students’ preferred specialties for residency programs. The correlation between academic achievement and preferred specialties among medical students provides valuable insights into the decision-making process. Even though academic performance played a significant role in students’ preferences for specialties, other factors were also important to consider. Medical schools and healthcare systems can better support the aspirations and needs of medical students by recognizing these factors and their potential impacts on medical education and future health. The importance of providing medical students with career guidance and counseling services was crucial to their future career choices.

## Data Availability

All datasets used and/or analyzed in this study are available upon request from the corresponding author.

## Abbreviations

USA: United States of America
PC: Primary care
GPA: Grade Point Average
OB/GYN: Obstetrics and Gynaecology
KSU: King Saud University
IRB: Institutional review board
SPSS: Statistical package for the social sciences
